# Reply: Koilocytes indicate a role for human papilloma virus in breast cancer

**DOI:** 10.1038/sj.bjc.6605550

**Published:** 2010-01-26

**Authors:** W K Glenn, B Heng, N J Whitaker, J S Lawson

**Affiliations:** 1 School of Biotechnology and Biomolecular Sciences, University of New South Wales, Sydney, Australia

**Sir,**

We reported the unambiguous presence of human papilloma virus (HPV) type 18 and HPV-associated koilocytes in breast cancers that had occurred in Australian women (Heng *et al*, 2009; Lawson *et al*, 2009). Koilocytes are epithelial cells characterised by perinuclear haloes surrounding condensed nuclei and are specifically associated with HPV infections and early oncogenic processes.

Sandstrom (2009) has made several interesting and important comments about these reports, namely: (i) he and his colleagues had previously made similar observations of HPV-associated koilocytes in breast cancer cases; (ii) Sarkola *et al* (2008) had identified HPV 16 in 4% of 223 human breast milk samples in Finland; and (iii) Cazzaniga *et al* (2009) had identified HPV of multiple types in 14% of 90 breast ductal lavages, HPV 16 in one of 10 breast colostrum samples, and HPV 5 and 23 in two of 25 breast milk samples in Italy.

We have also identified high-risk HPV (type 16) in human breast milk from normal unselected women. We used standard and *in situ* PCR techniques with identical primers and methods as reported in Heng *et al* (2009) to seek to identify HPV in breast milk. Using standard PCR, we screened DNA extracted from 24 samples of human breast milk from unselected normal (no history of breast cancer) women. Two (8.3%) were positive, both of whom were HPV type 16 as demonstrated by automated sequencing. In a different set of milk samples, using *in situ* PCR, we demonstrated HPV 16 in the nuclei of epithelial cells of one sample. This latter observation indicated that the outcomes based on standard PCR were unlikely to be due to contamination.

The implication of these various observations, including the identification of high-risk HPV in the Australian milk samples, is suggestive of a possible causal role of high-risk HPV in some breast cancers.
